# Theory of Mind and Information Processing Speed in Multiple Sclerosis: Associations With Personality Traits

**DOI:** 10.1002/brb3.71592

**Published:** 2026-07-09

**Authors:** Omid Mirmosayyeb, Aynaz Mohammadi, Saeed Vaheb, Mohammad Mohammadi, Aysa Shaygannejad, Jacob Balconi, Mohammad Yazdan Panah, Vahid Shaygannejad

**Affiliations:** ^1^ Isfahan Neurosciences Research Center Isfahan University of Medical Sciences Isfahan Iran; ^2^ Department of Neurology, School of Medicine Isfahan University of Medical Sciences Isfahan Iran; ^3^ School of Medicine Iran University of Medical Sciences Tehran Iran; ^4^ Department of Neurology, Jacobs Comprehensive MS Treatment and Research Center, Jacobs School of Medicine and Biomedical Sciences University At Buffalo, State University of New York Buffalo New York USA

**Keywords:** cognition, information processing speed, multiple sclerosis, personality, social cognition

## Abstract

**Background:**

Cognitive impairment, particularly decreased information processing speed (IPS), is a common symptom of multiple sclerosis (MS). Social cognition (SC) is also affected, but it is less studied, and the influence of personality traits on theory of mind (ToM), a key component of SC, and IPS remains unclear. Therefore, this study examined how IPS and ToM are associated with personality traits, demographic factors, and clinical factors in people with MS (PwMS).

**Methods:**

This cross‐sectional study included 100 PwMS attending a tertiary MS clinic in Isfahan, Iran. Demographic (age, sex, education, employment, marital status) and clinical data (disease type, duration, disability, and treatment) were collected. Personality traits were evaluated using the NEO Five‐Factor Inventory (FFI), ToM using the Reading the Mind in the Eyes Test (RMET), and IPS using the Symbol Digit Modalities Test (SDMT). Spearman correlations and linear regression analysis were used to examine associations.

**Results:**

The SDMT was correlated positively with the RMET (*r* = 0.35, *p* < 0.001) and Conscientiousness (*r* = 0.31, *p* = 0.001) and negatively with Expanded Disability Status Scale (EDSS) (*r* = −0.33, *p* < 0.001). The RMET was correlated with Extraversion (*r* = 0.31, *p* = 0.002), Agreeableness (*r* = 0.31, *p* = 0.002), Conscientiousness (*r* = 0.35, *p* < 0.001), and Openness (*r* = 0.40, *p* < 0.001). In multivariate regression analyses, the SDMT was associated with higher RMET (*B* = 0.37, *p* = 0.009) and lower EDSS (*B* = −1.98, *p* = 0.004). The RMET was associated with Openness (*B* = 0.43, *p* < 0.001), Agreeableness (*B* = 0.17, *p* = 0.020), and Conscientiousness (*B* = 0.18, *p* = 0.006).

**Conclusion:**

ToM was associated with personality traits, while IPS was associated with ToM performance and disability. Taken together, these cross‐sectional associations suggest that cognitive functioning in MS may relate to both brain‐ and person‐related factors.

## Graphical abstract

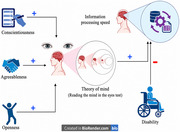

## 1 Introduction

Multiple sclerosis (MS) is a chronic, progressive neurodegenerative disorder that causes various neurological problems (Thompson et al. 2018), such as cognitive impairment (40%) (Askari et al. 2024; Benedict et al. 2020), depression (27%) (Peres et al. 2022; Patten et al. 2003), and fatigue (60%) (Yi et al. 2024; Penner and Paul 2017). Cognitive impairment can impact information processing speed (IPS), executive functions, social cognition (SC), perceptual‐motor skills, working memory, language, and sustained attention in people with MS (PwMS) (Olek 2021).

Among the cognitive domains affected by MS, IPS is the most commonly impaired. This impairment in IPS is often more marked than other cognitive deficits, such as language difficulties, which tend to be less frequent and less severe than IPS deficits (DeLuca et al. 2004; Forn et al. 2008). Furthermore, this impairment is more pronounced in progressive forms of MS than in relapsing‐remitting MS (RRMS) (Benedict et al. 2020; DeLuca et al. 2004; Forn et al. 2008; Johnen et al. 2017; De Sonneville et al. 2002). The crucial role of IPS in PwMS has been emphasized by numerous studies, which have shown its strong impact on working memory (Forn et al. 2008; Kouvatsou et al. 2022) and new learning skills (Chiaravalloti et al. 2013). Giving patients more time to process information has been shown to improve their working memory and planning skills (Leavitt et al. 2011; Owens et al. 2013), highlighting the critical role that IPS plays in overall cognitive functioning. Daily activities, such as employment, social interaction, and general quality of life, are also greatly impacted by decreased IPS (Benedict et al. 2005; Strober et al. 2014). The most common and sensitive test for assessing IPS in MS is the Symbol Digit Modalities Test (SDMT) (Benedict et al. 2017; Rao et al. 2017).

While IPS has been extensively studied, SC is a noteworthy yet relatively understudied aspect of cognitive functioning in MS. SC refers to the cognitive processes that support social interactions, including the ability to identify, interpret, and respond to others' intentions, attitudes, and behaviors. This definition distinguishes SC from broader cognitive abilities, such as executive functions, memory, and attention, which are often impaired in people with cognitive impairments (Adolphs 2001). The key components of SC are Theory of Mind (ToM), which is the ability to attribute mental states to oneself and others, empathy (the capacity to understand and share another person's feelings), social perception (the ability to interpret social cues and contexts), and emotion recognition (the ability to recognize and react to emotions). They are essential for social and communication functioning. In individuals with neurological disorders, these processes can have a substantial impact on social relationships and quality of life when they are impaired (Henry et al. 2009; Baron‐Cohen et al. 2001; Pinkham et al. 2016). Either ToM tasks or emotion recognition tasks are commonly used to evaluate SC (Henry et al. 2015). One of the most widely used tests of ToM is the Reading the Mind in the Eyes Test (RMET) (Baron‐Cohen et al. 2001). The RMET is considered to require comparatively less cognitive effort than other SC tasks (Henry et al. 2015). In the present study, SC is operationalized using the RMET; therefore, our SC findings primarily reflect ToM‐related performance rather than all facets of SC.

In addition to cognitive domains, personality traits have been shown to shape how PwMS cope with cognitive and social challenges (Maggio et al. 2020). A personality trait is defined as a consistent pattern of thoughts, emotions, and behaviors that differentiates individuals from one another (Maggio et al. 2020; Davidescu et al. 2021). The NEO Five‐Factor Inventory (NEO‐FFI) is one of the personality trait assessment tools that measures five dimensions: Neuroticism (tendency toward negative affect and emotional instability), Extraversion (sociability and positive emotionality), Openness (curiosity and preference for novelty and cognitive flexibility), Agreeableness (cooperativeness, trust, and empathy), and Conscientiousness (organization, self‐discipline, and goal‐directedness) (Costa 1992). PwMS are often described as having higher levels of Neuroticism and lower levels of Extraversion, Conscientiousness, and Agreeableness compared with healthy controls (Maggio et al. 2020; Davidescu et al. 2021; Jacot de Alcântara et al. 2023; Mirmosayyeb et al. 2025). The Findings from large cohort studies in PwMS show that greater Extraversion is associated with better cognitive performance, while higher Neuroticism is linked to a greater risk of memory impairments (Kever et al. 2022), anxiety, and depressive symptoms (Vaheb et al. 2024). Studies have suggested that Openness may act as a facet of cognitive reserve, protecting against cognitive decline (Benedict et al. 2020). Similarly, Conscientiousness has been associated with better cognitive outcomes and slower cognitive decline in patients with neurological conditions, including MS (Patten et al. 2003; Penner and Paul 2017). Some studies found that high Neuroticism and low Conscientiousness were linked to cognitive issues (Thompson et al. 2018), while other studies found that higher Openness and lower Neuroticism were linked to better memory (Kurtzke 1983). Differences in study design, the diversity of cognitive domains assessed, and sample sizes could explain these varied findings. Furthermore, Roy et al. showed that PwMS had higher Neuroticism and lower Extraversion compared with healthy controls, but reduced Conscientiousness was seen only in cognitively impaired patients. Importantly, Conscientiousness remained the key trait distinguishing impaired from unimpaired patients, even after controlling for demographics, disease factors, and depression (Roy et al. 2018). Another study by Fuchs et al. demonstrated in a large longitudinal cohort of PwMS that higher baseline Conscientiousness predicted a slower decline in SDMT scores over time (Fuchs 2020). These links are important because personality can affect thinking skills in different ways. For example, high Neuroticism may lead to emotional distractions, making it harder to process information. On the other hand, traits such as Openness and Conscientiousness can help people use better strategies to protect their brains from decline.

Nevertheless, personality traits have been examined in relation to cognitive and psychosocial outcomes in MS, but the results remain inconsistent. Specific domains such as IPS and ToM, a key component of SC, remain insufficiently documented. Although IPS and ToM are each independently linked to functional and social outcomes (Benedict et al. 2005, Strober et al. 2014; Henry et al. 2009; Baron‐Cohen et al. 2001; Pinkham et al. 2016), they are rarely examined together in the same MS sample, particularly when considering the role of personality. A better understanding of these associations may help explain heterogeneity in social and daily functioning impairments among patients. Therefore, this cross‐sectional study aimed to assess IPS using the SDMT and ToM using the RMET in PwMS and to examine their correlations with personality traits, demographics, and clinical characteristics.

## 2 Materials and Methods

### 2.1 Study Population and Design

This cross‐sectional study involved 100 PwMS in total. Participants in this study were all adult PwMS (age ≥18 years) who visited the tertiary MS clinic at Kashani Hospital, Isfahan, Iran, between August 2024 and February 2025 and who were diagnosed with MS according to the revised McDonald criteria (Thompson et al. 2018).

Any participant who displayed any of the following was excluded from the study: (1) major psychiatric disorder diagnosis (e.g., schizophrenia, bipolar disorder, or major depressive disorder); (2) alcohol or drug abuse during the past 12 months; (3) severe cognitive impairment, language barriers, or other disabilities that would prevent patients from understanding or completing study tests and questionnaires (as determined by the neurologist during the clinical evaluation); (4) psychotropic medication use (e.g., antipsychotics, benzodiazepines, or mood stabilizers); (5) corticosteroid medication use or clinical relapse in the last 30 days; (6) additional neurological conditions that impair cognition (e.g., vascular dementia, Alzheimer's disease); and (7) lack of informed consent. Every participant underwent a clinical neurological evaluation by a qualified neurologist to assess eligibility. Comorbid neurological disorders or any prior medical histories included in the exclusion criteria were found by reviewing the medical records.

The Ethics Committee of Isfahan University of Medical Sciences approved this study, which was carried out in compliance with the Declaration of Helsinki's ethical guidelines (IR.ARI.MUI.REC.1401.230). All eligible participants provided written informed consent.

### 2.2 Data Collection

The data were collected from medical records or by trained researchers under the supervision of a neurologist during routine visits. Participants' demographic information (age, sex, employment, marital status, and years of education) and disease characteristics (MS type, duration, functional status as determined by the Expanded Disability Status Scale [EDSS] (Kurtzke 1983) and disease‐modifying therapies (DMTs) received for MS were collected. The number of years of formal education (12 for high school, 16 for a bachelor's, 18 for a master's, and 22 for a PhD) was used to calculate educational level.

Cognitive function was assessed using two validated tools:

1. *The Symbol Digit Modalities Test*: IPS was assessed using the Persian version of the SDMT (Eshaghi et al. 2012). Within 90 s, participants were asked to verbally match symbols to the appropriate numbers as quickly and accurately as possible. The SDMT (Smith 1973) score ranges from 0 to 110, with higher scores indicating better IPS in the participant.
2. *The Reading the Mind in the Eyes Test*: ToM was evaluated using the Persian version of the RMET (Khorashad et al. 2015). The RMET, created by Baron‐Cohen et al. (2001) assesses the capacity to identify emotions by asking participants to choose the most appropriate mental state from four options while viewing images of different eye regions. The RMET score ranges from 0 to 36, with higher scores indicating better ToM performance of the participant.

The NEO‐FFI in Persian (Anisi 2012) was used to measure personality traits. Neuroticism, Extraversion, Openness, Agreeableness, and Conscientiousness are the five key personality domains that are assessed by the 60‐item NEO‐FFI self‐report questionnaire (Costa 1992).

### 2.3 Statistical Analysis

R version 4.2.2 and SPSS version 26 were used for the analyses. Numbers and percentages (%) were used to represent categorical variables, while the mean and standard deviation (SD) were used to represent continuous variables. The Kolmogorov–Smirnov test was used to assess whether the distributions of continuous variables were normal.

After observing non‐normality in most study variables, Spearman correlation coefficients were calculated to assess the bivariate associations between RMET, SDMT, personality traits, and all continuous demographic and clinical variables. The correlation analysis was conducted using the R package “Hmisc,” and the correlation matrix was visualized using the R package “ggplot2.”

To identify factors associated with the RMET and the SDMT, separate multivariate linear regression analyses were conducted using the enter method. Independent variables for each model were selected based on significant bivariate correlations (*p* < 0.05) with the respective dependent variable. The results of the regression models were reported as unstandardized beta coefficients (*B*), 95% confidence intervals (CIs), and *p* values.

Linear regression assumptions of linearity, homoscedasticity, and normality of residuals were assessed, and all were met for both multivariate regression analyses. Multicollinearity among independent variables in the multivariate regression models was assessed using variance inflation factors (VIFs), with values below 5 considered acceptable (Kim 2019).

A two‐tailed *p* value <0.05 was considered statistically significant in all analyses.

## 3 Results

### 3.1 Participants’ Characteristics

A total of 100 PwMS were included in the study. The mean age of participants was 36.2 years (SD = 6.2), and the majority were female (83%) and unemployed (59%). The mean duration of formal education was 14.6 years (SD = 2.7). Most participants had RRMS (91%), with a mean disease duration of 8.1 years (SD = 5.7) and a mean EDSS score of 1.9 (SD = 0.9) (Table 1).

**TABLE 1:** Characteristics of the participants.

Demographics	PwMS (*N* = 100)
Age, mean (SD)	36.2 (6.2)
Sex, n (%)	
Male	17 (17)
Female	83 (83)
Job, n (%)	
Unemployed	59 (59)
Employed	41 (41)
Marital status, n (%)	
Single	21 (21)
Married	76 (76)
Divorced	3 (3)
Education, mean (SD)	14.6 (2.7)
Disease characteristics	
MS type, n (%)	
RRMS	91 (91)
PPMS	4 (4)
SPMS	5 (5)
Disease duration, mean (SD)	8.1 (5.7)
EDSS, mean (SD)	1.9 (0.9)
Treatment, n (%)	
Rituximab	31 (31)
Ocrelizumab	20 (20)
Dimethyl fumarate	18 (18)
Teriflunomide	15 (15)
Interferon beta	12 (12)
Fingolimod	4 (4)
Personality traits, mean (SD)	
Neuroticism	25.4 (8.6)
Extraversion	25.8 (6.2)
Openness	25.1 (4.2)
Agreeableness	30.2 (5.4)
Conscientiousness	33.2 (7)
Cognition, mean (SD)	
SDMT	45.7 (6.3)
RMET	18 (4.4)

Abbreviations: EDSS, Expanded Disability Status Scale; MS, multiple sclerosis; PPMS, primary progressive MS; PwMS, people with multiple sclerosis; RMET, Reading the Mind in the Eyes Test; RRMS, relapsing‐remitting MS; SD, standard deviation; SDMT, Symbol Digit Modalities Test; SPMS, secondary progressive MS.

Regarding treatment, Rituximab (31%) and Ocrelizumab (20%) were the most commonly used DMTs. The mean scores for the cognitive and personality assessments were as follows: SDMT = 45.7 (SD = 6.3), RMET = 18.0 (SD = 4.4), Neuroticism = 25.4 (SD = 8.6), Extraversion = 25.8 (SD = 6.2), Openness = 25.1 (SD = 4.2), Agreeableness = 30.2 (SD = 5.4), and Conscientiousness = 33.2 (SD = 7) (Table 1).

Compared to recently published normative data from a demographically comparable Iranian population (healthy controls: mean age = 36.78 years, mean education = 14.43 years; SDMT mean = 51.24, SD = 9.77) (Habibi Moini et al. 2025), the present sample's mean SDMT of 45.7 was 0.57 standard deviations below the normative mean (*z*‐score = −0.57).

### 3.2 Correlations Between RMET, SDMT, Personality Traits, Demographic, and Clinical Characteristics

Spearman correlation analyses revealed that the SDMT had weak positive correlations with the RMET (*r* = 0.35, *p* < 0.001), Extraversion (*r* = 0.2, *p* = 0.046), Agreeableness (*r* = 0.27, *p* = 0.007), Conscientiousness (*r* = 0.31, *p* = 0.001), and education (*r* = 0.2, *p* = 0.042). The SDMT also had a weak negative correlation with EDSS (*r* = −0.33, *p* < 0.001).

The results showed that the RMET had weak positive correlations with Extraversion (*r* = 0.31, *p* = 0.002), Agreeableness (*r* = 0.31, *p* = 0.002), and Conscientiousness (*r* = 0.35, *p* < 0.001). The RMET also had a moderate positive correlation with Openness (*r* = 0.4, *p* < 0.001).

**FIGURE 1:**
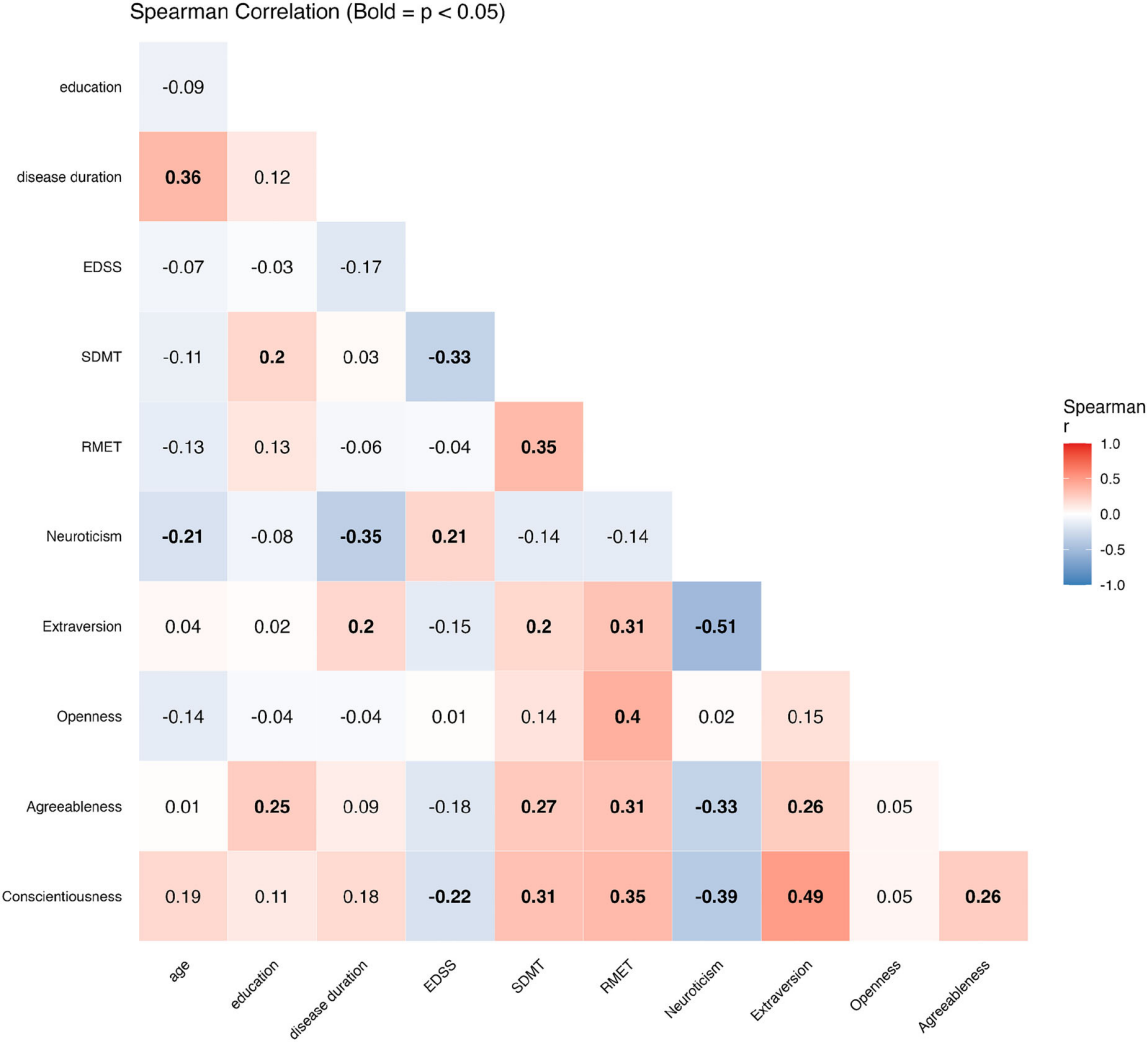
Correlations between the RMET (ToM), the SDMT, personality traits, demographic and clinical characteristics. Spearman correlation coefficients are presented, with statistically significant correlations (*p* < 0.05) shown in bold.

Other correlations are available in Figure 1, which shows the correlation matrix.

### 3.3 Factors Associated With RMET in PwMS

Variables that demonstrated significant Spearman correlations with RMET (Extraversion, Openness, Agreeableness, Conscientiousness, and SDMT) were entered into a multivariate linear regression model using the enter method. The overall model was significant (*F* = 12.68, *p* < 0.001, *R* ^2^ = 0.403). The results showed that Openness (*B* = 0.43, 95% CI: 0.26–0.60, *p* < 0.001), Agreeableness (*B* = 0.17, 95% CI: 0.03–0.31, *p* = 0.020), and Conscientiousness (*B* = 0.18, 95% CI: 0.05–0.30, *p* = 0.006) were significantly associated with the RMET (Table 2).

**TABLE 2:** Multivariate linear regression analysis assessing factors associations with RMET in PwMS.

	Multivariate		
	*B*	95% CI	*p* value
Extraversion	−0.02	−0.15, 0.12	0.805
Openness	0.43	0.26, 0.60	**<0.001**
Agreeableness	0.17	0.03, 0.31	**0.020**
Conscientiousness	0.18	0.05, 0.30	**0.006**
SDMT	0.08	−0.04, 0.21	0.176

Significant p‐values are in **bolds**. Abbreviations: *B*, unstandardized regression coefficient; CI, confidence interval; RMET, Reading the Mind in the Eyes Test; SDMT, Symbol Digit Modalities Test.

In the final multivariate regression model, all VIF values ranged between 1.06 and 1.54, indicating no evidence of problematic multicollinearity.

### 3.4 Factors Associated With SDMT in PwMS

Variables that demonstrated significant Spearman correlations with SDMT (Extraversion, Agreeableness, Conscientiousness, RMET, EDSS, and education) were entered into a multivariable linear regression model using the enter method. The overall model was significant (*F* = 5.51, *p* < 0.001, *R* ^2^ = 0.26). The results showed that higher EDSS (*B* = −1.98, 95% CI: −3.33 to −0.63, *p* = 0.004) was independently associated with lower SDMT scores, while RMET (*B* = 0.37, 95% CI: 0.09 to 0.64, *p* = 0.009) was positively associated with higher SDMT (Table 3).

**TABLE 3:** Multivariate linear regression analysis assessing factors associations with SDMT in PwMS.

	Multivariate		
	*B*	95% CI	*p* value
Education	0.27	−0.16, 0.70	0.213
EDSS	−1.98	−3.33, −0.63	**0.004**
Extraversion	−0.01	−0.22, 0.20	0.925
Agreeableness	0.07	−0.17, 0.30	0.579
Conscientiousness	0.14	−0.06, 0.34	0.158
RMET	0.37	0.09, 0.64	**0.009**

Significant p‐values are in **bold**.‐Abbreviations: *B*, unstandardized regression coefficient; CI, confidence interval; EDSS, Expanded Disability Status Scale; RMET, Reading the Mind in the Eyes Test; SDMT, Symbol Digit Modalities Test.

In the final multivariate regression model, all VIF values ranged between 1.08 and 1.62, indicating no evidence of problematic multicollinearity.

Although the SDMT was associated with the RMET in univariate analysis, it did not remain independently associated with the RMET in the multivariate model, whereas the RMET remained associated with the SDMT in the multivariate model predicting the SDMT.

## 4 Discussion

Our findings suggest that ToM in PwMS is related to personality traits, whereas IPS showed a different pattern of associations. In the fully adjusted multivariate regression models, clear patterns emerged for both cognitive outcomes. Individuals who scored higher on the personality traits of Openness, Agreeableness, and Conscientiousness all showed better ToM performance. On the other hand, reduced IPS was associated with greater neurological disability, while increased IPS was associated with better ToM performance.

Previous studies have also explored the link between SC and IPS in PwMS. Dulau et al. (2017) assessed SC in PwMS using the Bordeaux Social Cognition Evaluation Protocol, which evaluates multiple SC domains, including facial emotion recognition, ToM, emotional awareness, and cognitive and affective alexithymia, and found that 43% of patients were impaired on at least one SC test. In comparison, 20% showed impairments on two or more. Importantly, RMET scores were significantly lower in PwMS than in matched healthy controls and were positively correlated with executive function, working memory, attention, IPS, and episodic memory. Similarly, Raimo et al. (2017) also demonstrated that PwMS had impairments in both cognitive and affective ToM tasks, including the RMET, and their analysis revealed a strong correlation between RMET and SDMT scores. In line with these reports, our study also found a positive association between ToM performance and IPS, although the strength of this link was relatively modest. Neuroimaging studies have proposed potential neural substrates for this association. Evidence suggests that both SC and IPS rely on distributed neural networks, particularly the default mode, executive, and limbic networks, which are often disrupted in MS due to white matter damage and gray matter atrophy (Bisecco et al. 2020). Reduced functional connectivity within these networks has been associated with poorer SC performance as well as slower IPS (Meijer et al. 2018; Stampanoni Bassi et al. 2017). Key regions for SC, such as the amygdala, orbitofrontal cortex, and insula (Bisecco et al. 2020), may also be affected. Damage to these areas can slow the flow of information across neural pathways, which could subsequently impact IPS. These findings suggest that PwMS may have impairments in these domains because the brain circuits for SC and IPS overlap.

The association between RMET and SDMT observed in the multivariable model should be interpreted within the broader debate regarding whether social‐cognitive difficulties in MS represent relatively specific impairments or partially overlap with domain‐general cognitive dysfunction (Degraeve et al. 2024; Golde et al. 2020, Degraeve et al. 2026). In the present study, the relationship between the RMET and the SDMT suggests that mental‐state decoding may rely, at least in part, on information‐processing efficiency. This interpretation is consistent with previous evidence indicating that emotion‐recognition performance in MS may be associated with broader cognitive domains, including processing speed, executive functioning, and memory (Degraeve et al. 2026). However, the existing literature remains inconclusive. Some studies have suggested that social‐cognitive deficits may constitute partially distinct symptom profiles and may not merely reflect a secondary consequence of general cognitive impairment (Degraeve et al. 2026). This view is further supported by neuroimaging evidence demonstrating distinct functional connectivity patterns associated with impaired emotion recognition in MS (Golde et al. 2020). Therefore, our findings should not be interpreted as indicating that SC is reducible to processing speed. Rather, they suggest a partial overlap between social‐cognitive performance and domain‐general cognitive efficiency, while also acknowledging that social‐cognitive impairment in MS may involve partly specific mechanisms.

In further analyzing the variables affecting cognitive performance, we found that neurological disability has an impact on IPS in PwMS. In line with our findings, a diffusion tensor imaging study comparing 100 PwMS with 24 healthy controls highlighted this neurological burden; it found that only the EDSS and total white matter volume were significantly associated with SDMT scores (Grothe et al. 2022).

The association between the RMET and the SDMT should also be interpreted in light of the asymmetric findings across the two multivariate models. Although the SDMT was associated with the RMET in the bivariate analysis, it did not remain an independent predictor of RMET after personality traits were included in the model. In contrast, the RMET remained independently associated with the SDMT after adjustment for education, EDSS, and personality traits. This pattern should not be considered contradictory because regression models with different dependent variables and covariate structures are not statistically interchangeable. Instead, these findings suggest that the relationship between ToM and information processing speed is model‐dependent and may partly reflect shared variance with personality traits and neurological disability. Given the cross‐sectional design, these associations should not be interpreted as evidence of directionality or causality.

The findings of our study underscore the potential clinical utility of personality traits as protective factors in MS. Accordingly, developing personalized cognitive rehabilitation and support plans based on these traits may enhance the efficacy of interventions. Although our study does not establish causality, the results suggest that at‐risk patients may benefit most from interventions focusing on cognitive reserve, compensatory strategies, and SC, all of which have been shown to improve daily functioning (Benedict et al. 2020; Di Tella et al. 2023).

Using validated instruments to assess ToM and IPS, alongside personality traits, education, employment, and disability, in multivariate models is one of the study's strengths. We were able to identify independent effects in addition to correlations using this method. Nonetheless, several limitations must be noted. First, the cross‐sectional design avoids drawing conclusions regarding causality, specifically whether personality traits affect cognition or vice versa. Second, the majority of the participants were female, had RRMS, and had mild disabilities, and our sample size was small. These factors may have limited the generalizability of the findings and the ability to identify subtle associations. Third, neuroimaging data, which could have provided insight into the neural mechanisms underlying the observed associations, were not included. Fourth, despite the inclusion of important clinical and demographic factors, we failed to evaluate other possible confounders that may affect MS cognition and its association with personality traits, such as medication side effects, depression, or fatigue (Thelen et al. 2014; Guillemin et al. 2022; Diamond et al. 2008). The absence of assessments of mood symptoms, fatigue, and broader cognitive functions may have influenced the relationship among personality traits, employment status, and cognitive performance in PwMS. Fifth, the absence of culturally appropriate normative data for the Persian version of the RMET precluded a normative comparison for SC performance, and the development of such norms for Iranian populations represents an important direction for future research. Finally, multiple bivariate comparisons were conducted without a formal correction for multiplicity, thereby increasing the risk of type I error. While our key inferences are drawn from multivariate regression models rather than individual correlations, borderline associations should be interpreted with caution and require replication in future confirmatory studies. Given these limitations, future research should use longitudinal designs with larger and more varied sample sizes to ascertain the causal relationships between variables; incorporate neuroimaging to find brain networks connecting personality traits, IPS, and ToM; and concurrently measure mood and fatigue in addition to cognitive tests to give a more thorough understanding of the factors influencing cognitive performance in MS.

In summary, our research revealed that while IPS was independently associated with higher neurological disability and better ToM performance, ToM in PwMS is independently associated with higher scores in Openness, Agreeableness, and Conscientiousness. Taken together, these cross‐sectional associations suggest that cognitive functioning in MS may relate to both brain‐ and person‐related factors; however, no causal inferences can be drawn, and the observed association between ToM and IPS is modest, and both measures are likely to share variance with more general neuropsychological abilities. Therefore, future large‐scale longitudinal studies, ideally combining neuroimaging with comprehensive cognitive assessments that account for shared variance with general neuropsychological functions, are needed to clarify the temporal ordering and underlying mechanisms of these relationships, as well as their potential clinical applications.

## Author Contributions

**Omid Mirmosayyeb**: conceptualization, methodology, validation, supervision, project administration, writing – review and editing. **Saeed Vaheb**: data curation, writing – review and editing. **Aynaz Mohammadi**: validation, writing – original draft, writing – review and editing. **Aysa Shaygannejad**: data curation, writing – review and editing. **Mohammad Yazdan Panah**: investigation, writing – review and editing. **Vahid Shaygannejad**: conceptualization, investigation, validation, writing – review and editing. **Mohammad Mohammadi**: methodology, validation, formal analysis, writing – review and editing, writing – original draft. **Jacob Balconi**: conceptualization, investigation, validation, writing – review and editing.

## Funding

The authors have nothing to report.

## Ethics Statement

The Ethics Committee of Isfahan University of Medical Sciences approved this study, which was carried out in compliance with the Declaration of Helsinki's ethical guidelines (IR.ARI.MUI.REC.1401.230). All eligible participants provided written informed consent.

## Conflicts of Interest

The authors declare no conflicts of interest.

## Data Availability

The data supporting the findings of this study is available from the corresponding author upon a reseaonable request.
